# Agreement of Quantitative and Qualitative Antimicrobial Susceptibility Testing Methodologies: The Case of Enrofloxacin and Avian Pathogenic *Escherichia coli*

**DOI:** 10.3389/fmicb.2020.570975

**Published:** 2020-09-16

**Authors:** Robin Temmerman, Klara Goethals, An Garmyn, Gerty Vanantwerpen, Mia Vanrobaeys, Freddy Haesebrouck, Gunther Antonissen, Mathias Devreese

**Affiliations:** ^1^Department of Pharmacology, Toxicology and Biochemistry, Faculty of Veterinary Medicine, Ghent University, Merelbeke, Belgium; ^2^Department of Nutrition, Genetics and Ethology, Faculty of Veterinary Medicine, Ghent University, Merelbeke, Belgium; ^3^Department of Pathology, Bacteriology and Avian Diseases, Faculty of Veterinary Medicine, Ghent University, Merelbeke, Belgium; ^4^Animal Health Care Flanders, Torhout, Belgium

**Keywords:** antimicrobial resistance, antimicrobial susceptibility testing, avian pathogenic *Escherichia coli*, enrofloxacin, test agreement

## Abstract

Avian pathogenic *Escherichia coli* (APEC) is the causal agent of colibacillosis, one of the most common bacterial infections in the poultry sector. Antimicrobial susceptibility testing (AST) is essential for rational and prudent antimicrobial therapy. Subsequently, uniformity in test results from the various testing methodologies used in diagnostic laboratories is pivotal. The aim of this study was therefore to evaluate the agreement between different AST methods in determining fluoroquinolone resistance in APEC. Twenty APEC isolates were selected and subjected to four different susceptibility tests: the quantitative microbroth dilution, agar dilution and gradient strip tests, and the qualitative disk diffusion method. The experiments were performed in triplicate. Categorical agreement, essential agreement and different errors were assessed. Moreover, agreement was also evaluated by calculating intraclass correlation coefficients (ICCs) for the quantitative tests and determining the Pearson correlation coefficients for the agreement between the disk diffusion method and the quantitative tests. Categorical agreement and essential agreement when compared with the microbroth technique ranged from 85–95% and 85–100%, respectively. No very major errors (false susceptible) and only one major error (false resistant) and minor errors (results involving an intermediary category) were detected. The calculated ICC values of the three quantitative tests fluctuated around 0.970 (range 0.940–0.988). There was a high negative correlation between the disk diffusion method and the other tests (correlation coefficients ranging from −0.979 to −0.940), indicating a clear inverse relationship between the minimum inhibitory concentration value and the zone diameter of growth inhibition. In conclusion, the overall agreement between the four different testing methodologies was very high. These results confirm the reliability of the disk diffusion and gradient strip test methods as substantiated alternatives, next to the gold standard agar and microbroth dilution, for fluoroquinolone susceptibility testing of APEC isolates.

## Introduction

Colibacillosis is one of the major health threats in the poultry industry worldwide. This disease refers to any localized or systemic infection that is caused by the heterogeneous avian pathogenic *Escherichia coli* (APEC) pathotype (Nolan et al., 2020). This group of bacteria can act as both a primary and secondary infectious agent (Collingwood et al., 2014). A keyword to define APEC is diversity (Landman et al., 2014; Guabiraba and Schouler, 2015), summarizing their genomic heterogeneity and plasticity (Collingwood et al., 2014). Consequently, vaccination strategies, only generating serotype- and strain-specific protection (Kariyawasam et al., 2004; Dziva and Stevens, 2008), are insufficient to control this disease. This illustrates the need for different management measures and appropriate antimicrobial treatment. The fluoroquinolone class of antimicrobial drugs are frequently employed for this indication (Li et al., 2007; Persoons et al., 2012; Joosten et al., 2019).

Enrofloxacin (ENRO), first patented in 1984 (Trouchon and Lefebvre, 2016), is a second generation fluoroquinolone chemotherapeutic and is solely used in veterinary medicine. Enrofloxacin has two main targets in the bacterial cell, namely topoisomerase II (or DNA gyrase, main target in gram-negative bacteria) and topoisomerase IV (main target in gram-positive bacteria). These enzymes play a major role in the control of supercoiling processes of DNA and by extension in DNA transcription. Inhibition of these vital enzymes leads to a reduction in replicative activity (SOS response and cell filamentation) at low concentrations and quick cell death (chromosome fragmentation) at higher concentrations. This explains their dose dependent bacteriostatic and bactericidal activities (Drlica et al., 2008; Redgrave et al., 2014; Trouchon and Lefebvre, 2016).

The epidemiological link between antimicrobial usage and the development of antimicrobial resistance is unmistakable (Chantziaras et al., 2014). Emergence of antimicrobial resistance in APEC strains against ENRO and the fluoroquinolone class through (mis)usage is a major One Health concern, as this phenomenon both affects human (resistant zoonotic strains and transfer of antimicrobial resistance genes) and veterinary medicine (treatment failure and impaired animal welfare) (Moraru et al., 2012). The link with human medicine and their status of critical importance (WHO Advisory Group on Integrated Surveillance of Antimicrobial Resistance (AGISAR), 2019) are the major drivers for the increasing criticism of their use in veterinary medicine. Therefore it is imperative to use this class of antimicrobial agents judiciously in order to mitigate resistance development and dissemination, only treating with fluoroquinolones when the pathogen is determined susceptible. Decreased susceptibility against this class is predominantly the result of chromosomal single-step mutations in the genes coding for the main targets of these drugs (quinolone resistance determining regions, QRDR) (Temmerman et al., 2020).

Antimicrobial susceptibility testing (AST) is essential for rational antimicrobial drug usage and a mandatory condition to continue employing fluoroquinolones as treatment option in veterinary medicine in some countries (Royal, 2016; Van Driessche et al., 2018). Antimicrobial susceptibility testing can be performed either quantitatively or qualitatively. The qualitative disk diffusion (Kirby-Bauer) method is a relatively easy to perform technique routinely used in diagnostic laboratories. The main drawback of qualitative testing is the lack of a numerical minimum inhibitory concentration (MIC) value (only categorization as sensitive, intermediate, or resistant) and the possibility of large variations in the results (Liu et al., 2014). Quantitative testing methods provide numerical MIC values, which are more accurate descriptors of bacterial resistance levels. At present, the agar and microbroth dilution tests are regarded as the gold standard for quantitatively determining MIC values of different bacteria (Liu et al., 2014; Lallemand et al., 2016; Clinical and Laboratory Standards Institute, 2018; Van Driessche et al., 2018; Miftahussurur et al., 2020). However, these techniques are elaborate and require specialized equipment. The MIC-gradient strip test has gained acceptance as another quantitative method for susceptibility testing (Kelly et al., 1999), although it is not held in the same regard as the established agar and microbroth dilution methodologies (Van Driessche et al., 2018). However, there is consensus on the overall agreement between the strip test and the microbroth and agar dilution techniques for different “bug-drug” combinations (Jones et al., 1996; Hoogkamp-Korstanje et al., 1997; Kelly et al., 1999; Glupczynski et al., 2002; Liu et al., 2014; Deak et al., 2015). Procedures based on the strip test are more economical (lack of necessity of specialized equipment), less labor-intensive and quicker to perform (Chryssanthou and Cuenca-Estrella, 2002; Matar et al., 2003). However, the efficacy of the gradient strip test in AST of fluoroquinolones and APEC has not yet been investigated. Next to the paucity in gradient strip efficacy information, knowledge on the agreement between the other different AST methods is lacking for the specific APEC and ENRO combination. Most studies evaluating agreement between different AST methodologies have focused on human bacteria and fungi and antimicrobial agents frequently used in human medicine (Matar et al., 2003; Esteban et al., 2005; Rechenchoski et al., 2017).

Since different testing methodologies are performed in veterinary diagnostic laboratories, uniformity in susceptibility results from the different tests is crucial. In the present study, we evaluated the agreement between the MIC-gradient strip test and the more established microbroth and agar dilution tests together with the qualitative disk diffusion method for the evaluation of ENRO susceptibility or resistance for a collection of clinical APEC isolates.

## Materials and Methods

### Strains

*Staphylococcus aureus* ATCC 29213 and *E. coli* ATCC 25922 were used as quality control reference strains in all of the antimicrobial susceptibility tests.

Twenty strains were selected from our database of clinical APEC isolates previously obtained by Animal Health Care Flanders (Torhout, Belgium) and Sciensano (Brussels, Belgium). These were stored at approximately −70°C. Strains were selected based on earlier MIC results (determined by gradient strip test) in order to have a balance between wild type (WT, *n* = 11) and non-wild type strains (NWT, *n* = 9). The distinction between WT and NWT is based on the epidemiological cut off (ECOFF), which is 0.125 μg/mL for ENRO for *E. coli* (EUCAST, 2020).

### Antimicrobial Susceptibility Testing

One experiment consisted of the evaluation of the susceptibility of the twenty selected isolates together with the control strains using the four AST methodologies (gradient-strip test, microbroth dilution, agar dilution, and disk diffusion). The different tests were performed in triplicate on different occasions (three separate experiments).

#### MIC-Gradient Strip Test

The procedure was carried out as described previously (DeMars et al., 2016; Van Driessche et al., 2018). In brief, APEC strains were grown overnight on McConkey agar (Oxoid, Thermo Fisher Scientific, Merelbeke, Belgium). After incubation (37°C), several colonies (1–5) were added to a glass tube containing 3 mL sterile PBS and mixed in order to achieve a 0.5 McFarland inoculum (∼1.5 × 10^8^ colony forming units (cfu)/mL) (ATB 1550 densitometer, Biomerieux, Schaerbeek, Belgium). Next, using a sterile cotton swab, a homogenous bacterial lawn (approximately 100 μL) was streaked onto Mueller Hinton (MH) agar plates (BD BBL^TM^, Thermo Fisher Scientific, Merelbeke, Belgium). Finally, the MIC test strips (Liofilchem s.r.l., Roseto degli Abruzzi, Italy) were placed at the center of the plate and incubated for approximately 24 h at 37°C. Afterward, the results were read and recorded. This was done was by evaluating the ellipsoid zones of bacterial growth inhibition and examining the intersection of this zone and the concentration mark of the test strip, which indicated the MIC. To comply with the standard doubling dilutions, the in-between results were rounded up to the next upper two-fold value (e.g., 0.023 μg/mL was rounded up to 0.032 μg/mL).

#### Microbroth Dilution Test

The technique was performed in accordance with CLSI standards (Clinical and Laboratory Standards Institute, 2018). From a 0.5 McFarland inoculum, 100 μL was taken and diluted 1:100 in 10 mL cation-adjusted Mueller Hinton broth (CAMHB) (BD BBL^TM^, Thermo Fisher Scientific, Merelbeke, Belgium). Next, 50 μL of the diluted inoculum was transferred to each well of a 96 well plate containing 50 μL of CAMHB with or without ENRO (1:2 dilutions), resulting in an inoculum size of ±5 × 10^5^ cfu/mL. Finally, the 96 well plates were tightly sealed with adhesive foil and stored in an incubator for approximately 24 h at 37°C.

#### Agar Dilution Test

The test was carried out in compliance with EUCAST standards (EUCAST, 2020). The 0.5 McFarland inoculum was diluted 1:10 in sterile PBS and 1 μL of the dilution was spotted on the MH agar plates supplemented with different ENRO concentrations (ranging from 0.004 to 32 μg/mL in two-fold increases), resulting in a final concentration of 10^4^ cfu/mL per spot. Following incubation (24 h, 37°C), the MIC was interpreted as the agar plate where there was no longer bacterial growth (growth inhibition).

#### Disk Diffusion Test

The procedure was carried out in accordance to CLSI standards (Clinical and Laboratory Standards Institute, 2018). Similar to the MIC-gradient strip test, a bacterial lawn of approximately 100 μL was uniformly streaked on MH agar plates from a 0.5 McFarland inoculum prepared in sterile PBS. The ENRO disks (10 μg, Rosco Diagnostica A/S, Taarstrup, Denmark) were placed on the agar and subsequently incubated for approximately 24 h in ambient air (37°C). Following incubation, the circular growth inhibition zones (in millimeters, mm) were measured with a manual calliper.

### Clinical Breakpoints

Strains were designated as susceptible (S), intermediate (I), and resistant (R) based on their respective MIC values or the mm measurements and the CLSI-defined interpretive criteria (Clinical and Laboratory Standards Institute, 2018; Table 1).

**TABLE 1:** Enrofloxacin interpretive criteria for (avian pathogenic) *Escherichia coli* as stated by CLSI.

	Interpretive criteria		
	Susceptible	Intermediate	Resistant
**Enrofloxacin**			
MIC, μg/mL	≤0.25	0.5–1	≥2
Disk diffusion (5 μg), mm	≥23	17–22	≤16

### Data Analysis

Multiple statistical approaches were used to assess the conformity of the different tests.

Based on the categorization of the strains into different susceptibility classes for the different tests, very major (VME), major (ME), and minor errors (mE) were calculated by using proportions (percent). VME, ME, and mE are defined as a false susceptible result, a false resistant result and a result involving an intermediate category, respectively (Thornsberry et al., 1980; Jorgensen, 1993; Deak et al., 2015). Essential agreement and categorical agreement were also assessed. Essential agreement was defined as an MIC value within a log_2_ dilution of the MIC result obtained from the microbroth dilution technique. Categorical agreement was defined as a S, I, or R interpretation that was conform the microbroth dilution result (Deak et al., 2015).

The agreement between the quantitative gradient strip, agar dilution and microbroth dilution tests was also evaluated through the intraclass correlation coefficient (ICC). Before analysis, the values of the MIC’s were log_2_ transformed. The ICC was based on a two-way mixed effects model (Koo and Li, 2016). In the model the log_2_ of the MIC score is the dependent variable, the sample is the random effect and the technique is the fixed effect. The ICC was calculated separately for each experiment.

Since disk diffusion is in another scale than the other three techniques (mm measurements), the ICC statistical technique cannot be used for this method. Instead, the Pearson correlation coefficient was calculated to describe the agreement between disk diffusion and the other techniques two by two and for each experiment separately.

Data analysis was done in R 4.0.0 [R Core Team (2019), Vienna, Austria] and GraphPad Prism version 8.3.0 for Windows (GraphPad Software, San Diego, CA, United States).

## Results

The results of the quality control bacteria for all the different tests were within the acceptable control ranges in accordance to the CLSI guidelines (Clinical and Laboratory Standards Institute, 2018), namely between 0.008–0.03 μg/mL and 0.06–0.25 μg/mL for *E. coli* ATCC 25922 and *S. aureus* ATCC 29213, respectively. The MIC values of the different clinical APEC isolates ranged from 0.008 to 32 μg/mL (results not shown).

The performance results of the gradient strip, agar dilution and disk diffusion test when compared with the microbroth dilution technique are listed in Table 2. The essential agreement between the gradient strip test and the microbroth dilution testing method was 100% in the three experiments. For the agar dilution method, essential agreement ranged from 85 to 100%. According to the microbroth procedure, 12 strains were considered S, 3 I, and 5 R. This was similar over the three experiments. Using disk diffusion as categorization measure, 13 strains were S, 3 I, and 4 R. Again, the same result was obtained during the three experiments. Categorical agreement between the microbroth dilution technique and the disk diffusion test ranged from 85 to 90%. In 2 experiments, the 20 strains were identified as 13 S, 2 I, and 5 R according to the gradient strip test, while in one experiment this was 12 S, 2 I, and 6 R. Categorical agreement between this technique and the reference microbroth method ranged from 85 to 95% over the three experiments. Finally, the agar dilution technique classified the strains as 11 S, 3 I, and 6 R in two experiments and 12 S, 2 I, and 6 R in one experiment with a categorical agreement with the microbroth method of 95% in all experiments.

**TABLE 2:** Performances of gradient strip test, agar dilution and disk diffusion compared to the microbroth dilution technique for enrofloxacin susceptibility testing in avian pathogenic *Escherichia coli* in different experiments.

**Experiment 1**									
	**No. of isolates^a^**				**Performance [no. (%)]^b^**				
**Method**	**Total**	**S**	**I**	**R**	**EA**	**CA**	**mE**	**ME**	**VME**

Microbroth dilution	20	12	3	5	NA	NA	NA	NA	NA
Strip test	20	13	2	5	100	95	5	0	0
Agar dilution	20	12	2	6	100	95	5	0	0
Disk diffusion	20	13	4	3	NA	90	10	0	0
**Experiment 2**									
	**No. of isolates**				**Performance [no. (%)]**				
**Method**	**Total**	**S**	**I**	**R**	**EA**	**CA**	**mE**	**ME**	**VME**

Microbroth dilution	20	12	3	5	NA	NA	NA	NA	NA
Strip test	20	13	2	5	100	95	5	0	0
Agar dilution	20	11	3	6	90	95	0	5	0
Disk diffusion	20	13	4	3	NA	90	10	0	0
**Experiment 3**									
	**No. of isolates**				**Performance [no. (%)]**				
**Method**	**Total**	**S**	**I**	**R**	**EA**	**CA**	**mE**	**ME**	**VME**

Microbroth dilution	20	12	3	5	NA	NA	NA	NA	NA
Strip test	20	12	2	6	100	85	15	0	0
Agar dilution	20	11	3	6	85	95	5	0	0
Disk diffusion	20	13	4	3	NA	85	15	0	0

*^*a*^S, susceptible; I, intermediate; R, resistant. ^*b*^EA, essential agreement; CA, categorical agreement; mE, minor error; ME, major error; VME, very major error; NA, not applicable.*

No VMEs were detected in the three experiments. Only one ME was detected, when comparing the agar dilution method with the microbroth dilution test. A strain reported as susceptible in the latter (0.25 μg/mL) had a MIC value of 2 μg/mL that corresponded with the category for resistance in the former. Eight of the nine comparisons with the microbroth dilution test showed mEs. The frequency ranged from 5 to 15% across the experiments. On average, the disk diffusion method had the highest number of mEs (11.7%), followed by the gradient (8.3%) and agar dilution tests (5%).

**FIGURE 1:**
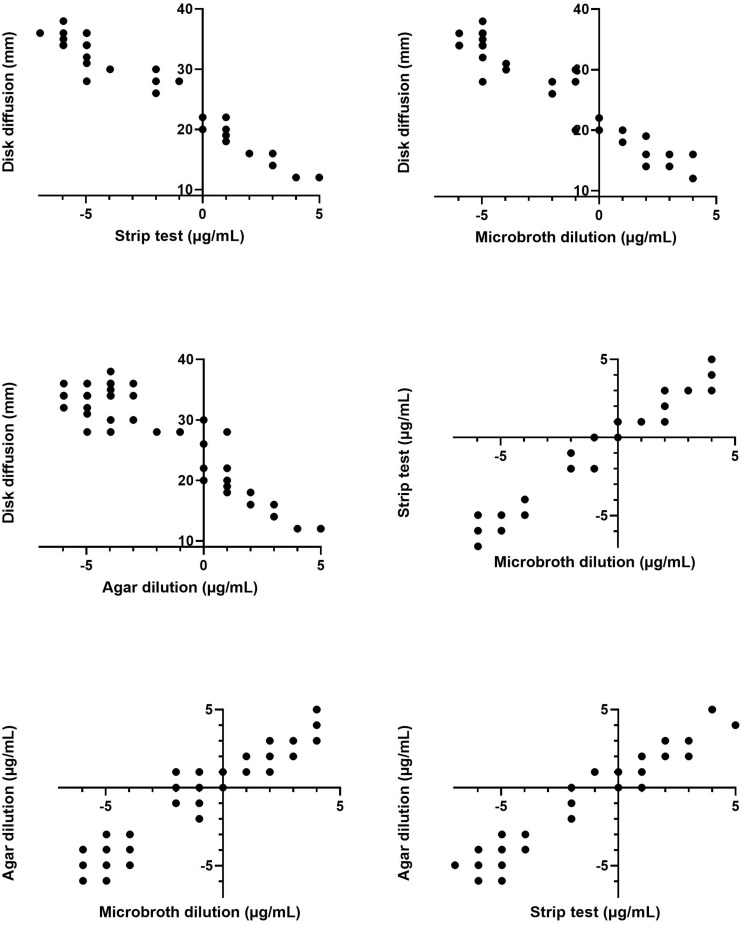
Scatterplots of the pairwise comparisons of the aggregated data (of the three experiments) of the four antimicrobial susceptibility tests. MIC values determined via the gradient strip, agar dilution and microbroth dilution tests are log_2_ transformed. Note that one data point can correspond with more than one result.

Figure 1 presents the scatterplots of the data combined from the three experiments. Six pairwise comparisons between the results of four different tests were made. As can be derived from visual inspection of the plots, there is a strong positive trend between the different quantitative tests. Oppositely, the relationship between the disk diffusion method and the other testing methodologies is strongly negative.

The calculated ICC value of the three quantitative tests [95% confidence interval] for the first experiment was 0.967 [0.940;0.984]. The ICC values for the second and third experiment were 0.976 [0.956;0.988] and 0.975 [0.955;0.988].

The determined Pearson correlation coefficients of the three pairwise comparisons between the disk diffusion and the other quantitative tests are listed in Table 3. In general, there was a very high negative correlation, irrespective of test type or experiment. The correlation coefficients ranged from -0.979 to -0.940.

**TABLE 3:** Overview of the calculated Pearson correlation coefficients [95% confidence intervals] of the pairwise comparisons between disk diffusion and the other techniques for enrofloxacin susceptibility testing in avian pathogenic *Escherichia coli* for each experiment separately.

	Pearson correlation coefficient		
	Experiment 1	Experiment 2	Experiment 3
DD–ST	−0.979	−0.943	−0.952
	[−0.992; −0.947]	[−0.978; −0.859]	[−0.981; −0.881]
DD–AD	−0.979	0.954	−0.965
	[−0.992; −0.946]	[−0.982; −0.884]	[−0.986; −0.912]
DD–MD	−0.967	−0.940	−0.968
	[−0.987; −0.917]	[−0.976; −0.851]	[−0.988; −0.920]

*DD, disk diffusion; ST, gradient strip test; AD, agar dilution; MD, microbroth dilution.*

## Discussion

Antimicrobial susceptibility testing of bacteria associated with disease is essential for judicious and rational antimicrobial treatment. However, several susceptibility testing methodologies are available and used by different (veterinary) diagnostic laboratories. Consistency between the results of the different tests is essential, as variability in MIC values or in susceptibility categorization can have a major impact on the choice of treatment by the clinician and subsequently on patient (animal) welfare and morbidity.

In this study, the agreement between four frequently used AST techniques was investigated. Overall, inter-test agreement was very high. No VMEs were detected in all experiments. Essential agreement between the gold standard microbroth dilution and the gradient strip test was 100%, meaning that the MIC value obtained by the strip test was always within a log2 dilution of the MIC result obtained from the microbroth dilution technique. Despite 100% essential agreement, categorical agreement fluctuated between 85 and 95%. An explanation for the difference between essential and categorical agreement can be deducted to the APEC strains with MIC values that border a clinical breakpoint (Table 1). A strain with an MIC value of 0.25 μg/mL (which is the clinical breakpoint) in one test is categorized as susceptible. When another test finds a MIC value of 0.5 μg/mL, the strains is regarded as intermediate. Despite the essential agreement (result was within a log_2_ dilution of the MIC result of the other test), the same strain was classified differently in the two tests.

It is paramount for a quantitative AST system to generate reproducible results. According to Jorgensen (1993), a new, not standardized susceptibility testing method (1) should provide >90% agreement (within ±1 twofold dilution) with the MIC’s determined by the reference technique, (2) should contain less than 3% of VME, and (3) the combination of ME and mE should be below 7%. Notwithstanding the fact that the gradient strip test is no longer a novel technique, the gradient-strip test clearly met the above mentioned criteria, except for a slightly higher error prevalence. The average essential and categorical agreement was 100 and 88.3%, no VMEs were detected and the average combination of minor and major error was 8.3%. However, the marginally higher occurrence of mEs could be due to the small sample size (Jorgensen, 1993). Therefore, the MIC-gradient strip test can be regarded as a substantiated and valid alternative to the other quantitative gold standard methodologies with additional advantages such as a reduction in time consumption, labor and consumables. This is in accordance with other studies evaluating the validity of the gradient strip test for fluoroquinolone AST with other bacteria involved in clinical infections in humans, such as *Salmonella enterica*, *Pseudomonas aeruginosa*, *Streptococcus pneumoniae* and *S. aureus* (Jones et al., 1996; Deak et al., 2015). Additional studies are desired to investigate the reliability of the gradient strip test for susceptibility testing of APEC isolates to other antimicrobial drugs.

Agar dilution also showed high categorical and essential agreement when compared with the microbroth dilution technique. On average, this method had the lowest occurrence of mE’s (5%). One ME was detected when using this technique, meaning that a strain was falsely classified as resistant while it was evaluated as susceptible by the microbroth test. The performance of the qualitative disk diffusion method dovetails with the aforementioned quantitative tests. Categorical agreement was on average 88.3%, which was slightly lower than for the other tests (91.7% for the strip test and 95% for agar dilution test). Essential agreement could not be evaluated since no numerical MIC values were determined. On par with the lower categorical agreement, the prevalence of mE’s was higher than the other tests (11.7%). In contrast with some studies investigating different bacterial strains and antimicrobial agents (Biedenbach et al., 1993; Lehtopolku et al., 2012; Rechenchoski et al., 2017), the results of this study strengthen the validity of using the disk diffusion method for identifying resistance of APEC strains.

Agreement was also evaluated by determining the ICC value between the different quantitative tests. The ICC is a measure of test-retest, intrarater and interrater or inter-test reliability (Koo and Li, 2016). Reliability is defined as the extent to which measurements can be replicated (Bruton et al., 2000; Koo and Li, 2016). Several ICC forms are available (Shrout and Fleiss, 1979; McGraw and Wong, 1996; Koo and Li, 2016). In this study, the ICC based on a two-way mixed effects model, single rater/measurement and focus on consistency was chosen. This measure is termed ICC (3,1) according to the Shrout and Fleiss convention (Shrout and Fleiss, 1979). The ICC (3,1) values over the three experiments (ranging from approximately 96–98%) were decidedly high and the ranges of 95% confidence intervals were very narrow varying from 0.033 to 0.044. Based on the 95% confidence intervals, the reliability and agreement level can be interpreted as excellent (lower and upper bounds >0.9) (Koo and Li, 2016).

As stated earlier, the disk diffusion method was not included in the ICC analysis because of differences in measurement scale (mm versus μg/mL). Instead, the correlation between disk diffusion and the other three quantitative tests was assessed. The negative correlation between disk diffusion and the three quantitative methods was very high (Mukaka, 2012). The Pearson correlation coefficients, ranging from -0.979 to -0.940, were comparable between the different techniques and showed little variability between experiments. This strongly negative relationship is logical as a higher MIC value is associated with strains with reduced susceptibility, which in turn leads to smaller growth inhibition zones and smaller mm values.

In conclusion, these findings demonstrate the consistency and reliability of the results obtained via the different AST methods for APEC and ENRO. The three quantitative MIC testing methods showed very high agreement (essential and categorical). This demonstrates that the gradient strip test is a valid alternative for the current gold standard microbroth and agar dilution tests for detecting fluoroquinolone resistance in *E.coli.* Additionally, the present study illustrates the superb reliability of the disk diffusion test for (categorical) fluoroquinolone susceptibility testing in APEC. Results obtained through either of the methodologies provide uniform results which should guide poultry veterinarians in choosing the same evidence-based treatment option in all cases.

## Data Availability Statement

The raw data supporting the conclusions of this article will be made available by the authors, without undue reservation.

## Author Contributions

RT conceived and designed the study and performed the bacteriological experiments. KG and RT performed the data analysis. RT wrote the first draft of the manuscript. All authors critically reviewed several drafts of the manuscript.

## Conflict of Interest

The authors declare that the research was conducted in the absence of any commercial or financial relationships that could be construed as a potential conflict of interest.

## References

[B1] Biedenbach D. J. Jones R. N. Erwin M. E. (1993). Interpretive accuracy of the disk diffusion method for testing newer orally administered cephalosporins against *Morganella morganii*. *J. Clin. Microbiol.* 31 2828–2830. 10.1128/jcm.31.10.2828-2830.1993 8253998 PMC266029

[B2] Bruton A. Conway J. H. Holgate S. T. (2000). Reliability: what is it, and how is it measured? *Physiotherapy* 86 94–99. 10.1016/S0031-9406(05)61211-4

[B3] Chantziaras I. Boyen F. Callens B. Dewulf J. (2014). Correlation between veterinary antimicrobial use and antimicrobial resistance in food-producing animals: a report on seven countries. *J. Antimicrob. Chemother.* 69 827–834. 10.1093/jac/dkt443 24216767

[B4] Chryssanthou E. Cuenca-Estrella M. (2002). Comparison of the antifungal susceptibility testing subcommittee of the European committee on antibiotic susceptibility testing proposed standard and the E-test with the NCCLS broth microdilution method for voriconazole and caspofungin susceptibility testing of yeast species. *J. Clin. Microbiol.* 40 3841–3844. 10.1128/JCM.40.10.3841-3844.2002 12354895 PMC130859

[B5] Clinical and Laboratory Standards Institute (2018). *VET08: Performance Standards for Antimicrobial Disk and Dilution Susceptibility Test for Bacteria Isolated from Animals*, 4 Edn Wayne, PA: Clinical and Laboratory Standards Institute.

[B6] Collingwood C. Kemmett K. Williams N. Wigley P. (2014). Is the concept of avian pathogenic *Escherichia Coli* as a single pathotype fundamentally flawed? *Front. Vet. Sci.* 1:5. 10.3389/fvets.2014.00005 26664913 PMC4668852

[B7] Deak E. Hindler J. A. Skov R. Sjölund-Karlsson M. Sokovic A. Humphries R. M. (2015). Performance of etest and disk diffusion for detection of ciprofloxacin and levofloxacin resistance in *Salmonella Enterica*. *J. Clin. Microbiol.* 53 298–301. 10.1128/JCM.02715-14 25355768 PMC4290941

[B8] DeMars Z. Biswas S. Amachawadi R. G. Renter D. G. Volkova V. V. (2016). Antimicrobial susceptibility of enteric gram negative facultative anaerobe bacilli in aerobic versus anaerobic conditions. *PLoS One* 11:e155599. 10.1371/journal.pone.0155599 27191612 PMC4871507

[B9] Van Driessche L. Bokma J. Gille L. Ceyssens P. J. Sparbier K. Haesebrouck F. (2018). Rapid detection of tetracycline resistance in bovine pasteurella multocida isolates by MALDI biotyper antibiotic susceptibility test rapid assay (MBT-ASTRA). *Sci. Rep.* 8 1–10. 10.1038/s41598-018-31562-8 30206239 PMC6134125

[B10] Drlica K. Malik M. Kerns R. J. Zhao X. (2008). Quinolone-mediated bacterial death. *Antimicrob. Agents Chemother.* 52 385–392. 10.1128/AAC.01617-06 17724149 PMC2224783

[B11] Dziva F. Stevens M. P. (2008). Colibacillosis in poultry: unravelling the molecular basis of virulence of avian pathogenic *Escherichia Coli* in their natural hosts. *Avian Pathol.* 37 355–366. 10.1080/03079450802216652 18622850

[B12] Esteban A. Abarca M. L. Cabañes F. J. (2005). Comparison of disk diffusion method and broth microdilution method for antifungal susceptibility testing of dermatophytes. *Med. Mycol.* 43 61–66. 10.1080/13693780410001711972 15712608

[B13] EUCAST (2020). *Antimicrobial Wild Type Distributions of Microorganisms.* Available online at: https://mic.eucast.org/Eucast2/ (accessed May 13, 2020).

[B14] Glupczynski Y. Broutet N. Cantagrel A. Andersen L. Alarcon T. López-Brea M. (2002). Comparison of the E test and agar dilution method for antimicrobial suceptibility testing of *Helicobacter* pylori. *Eur. J. Clin. Microbiol. Infect. Dis.* 21 549–552. 10.1007/s10096-002-0757-6 12172749

[B15] Guabiraba R. Schouler C. (2015). Avian colibacillosis: still many black holes. *FEMS Microbiol. Lett.* 362 1–8. 10.1093/femsle/fnv118 26204893

[B16] Hoogkamp-Korstanje J. A. Dirks-Go S. I. Kabel P. Manson W. L. Stobberingh E. E. Vreede R. W. (1997). Multicentre in-vitro evaluation of the susceptibility of *Streptococcus* pneumoniae, *Haemophilus Influenzae* and *Moraxella* catarrhalis to ciprofloxacin, clarithromycin, co-amoxiclav and sparfloxacin. *J. Antimicrob. Chemother.* 39 411–414. 10.1093/jac/39.3.411 9096192

[B17] Jones R. N. Erwin M. E. Croco J. L. (1996). Critical appraisal of E test for the detection of fluoroquinolone resistance. *J. Antimicrob. Chemother.* 38 21–25. 10.1093/jac/38.1.21 8858453

[B18] Joosten P. Sarrazin S. Van Gompel L. Luiken R. E. C. Mevius D. J. Wagenaar J. A. (2019). Quantitative and qualitative analysis of antimicrobial usage at farm and flock level on 181 broiler farms in nine european countries. *J. Antimicrob. Chemother.* 74 798–806. 10.1093/jac/dky498 30649428

[B19] Jorgensen J. H. (1993). Selection criteria for an antimicrobial susceptibility testing system. *J. Clin. Microbiol.* 31 2841–2844. 10.1128/jcm.31.11.2841-2844.1993 8263164 PMC266141

[B20] Kariyawasam S. Wilkie B. N. Gyles C. L. (2004). Construction, characterization, and evaluation of the vaccine potential of three genetically defined mutants of avian pathogenic *Escherichia Coli*. *Avian Dis.* 48 287–299. 10.1637/7093 15283416

[B21] Kelly L. M. Jacobs M. R. Appelbaum P. C. (1999). Comparison of agar dilution, microdilution, etest and disc diffusion to test the activity of trovafloxacin against *Pseudomonas Aeruginosa*, methicillin-resistant *Staphylococcus* aureus and *Streptococcus* pneumoniae. *J. Antimicrob. Chemother.* 43 707–709. 10.1093/jac/43.5.707 10382894

[B22] Koo T. K. Li Y. M. (2016). A guideline of selecting and reporting intraclass correlation coefficients for reliability research. *J. Chiropr. Med.* 15 155–163. 10.1016/j.jcm.2016.02.012 27330520 PMC4913118

[B23] Lallemand E. A. Lacroix M. Z. Toutain P. L. Boullier S. Ferran A. A. Bousquet-Melou A. (2016). In vitro degradation of antimicrobials during use of broth microdilution method can increase the measured minimal inhibitory and minimal bactericidal concentrations. *Front. Microbiol.* 7:2051. 10.3389/fmicb.2016.02051 28066372 PMC5175475

[B24] Landman W. J. M. Buter G. J. Dijkman R. van Eck J. H. H. (2014). Molecular typing of avian pathogenic *Escherichia Coli* colonies originating from outbreaks of *E. Coli* peritonitis syndrome in chicken flocks. *Avian Pathol.* 43 345–356. 10.1080/03079457.2014.935291 24944080

[B25] Lehtopolku M. Kotilainen P. Puukka P. Nakari U. M. Siitonen A. Eerola E. (2012). Inaccuracy of the disk diffusion method compared with the agar dilution method for susceptibility testing of campylobacter Spp. *J. Clin. Microbiol.* 50 52–56. 10.1128/JCM.01090-11 22075583 PMC3256729

[B26] Li Q. Bi X. Diao Y. Deng X. (2007). Mutant-prevention concentrations of enrofloxacin for *Escherichia coli* isolates from chickens. *Am. J. Vet. Res.* 68 812–815. 10.2460/ajvr.68.8.812 17669019

[B27] Liu H. Taylor T. H. Pettus K. Trees D. (2014). Assessment of etest as an alternative to agar dilution for antimicrobial susceptibility testing of *Neisseria gonorrhoeae*. *J. Clin. Microbiol.* 52 1435–1440. 10.1128/JCM.02131-13 24554750 PMC3993651

[B28] Matar M. J. Ostrosky-Zeichner L. Paetznick V. L. Rodriguez J. R. Chen E. Rex J. H. (2003). Correlation between E-test, disk diffusion, and microdilution methods for antifungal susceptibility testing of fluconazole and voriconazole downloaded from. *Antimicrob. Agents Chemother.* 47 1647–1651. 10.1128/AAC.47.5.1647-1651.2003 12709335 PMC153338

[B29] McGraw K. O. Wong S. P. (1996). Forming inferences about some intraclass correlation coefficients. *Psychol. Methods* 1 30–46. 10.1037/1082-989X.1.1.30

[B30] Miftahussurur M. Fauzia K. A. Nusi I. A. Setiawan P. B. Syam A. F. Waskito L. A. (2020). E-test versus agar dilution for antibiotic susceptibility testing of *Helicobacter* pylori: a comparison study. *BMC Res. Notes* 13:9. 10.1186/s13104-019-4877-9 31924273 PMC6954499

[B31] Moraru R. Pourcher A.-M. Jadas-Hecart A. Kempf I. Ziebal C. Kervarrec M. (2012). Changes in concentrations of fluoroquinolones and of ciprofloxacin-resistant *Enterobacteriaceae* in chicken feces and manure stored in a heap. *J. Environ. Qual.* 41, 754–763. 10.2134/jeq2011.0313 22565257

[B32] Mukaka M. M. (2012). Statistics corner: a guide to appropriate use of correlation coefficient in medical research. *Malawi Med. J.* 24 69–71. 23638278 PMC3576830

[B33] Nolan L. K. Vaillancourt J. P. Barbieri N. L. Logue C. M. (2020). “Colibacillosis,” in *Diseases of Poultry*, 14 Edn, eds Suarez D. L. Swayne D. E. Boulianne M. Logue C. M. McDougald L. R. Nair V. (Iowa, IA: Iowa State Press), 770–830.

[B34] Persoons D. Dewulf J. Smet A. Herman L. Heyndrickx M. Martel A. (2012). Antimicrobial use in belgian broiler production. *Prevent. Vet. Med.* 105 320–325. 10.1016/j.prevetmed.2012.02.020 22459488

[B35] R Core Team (2019). *R: A Language and Environment for Statistical Computing*. Vienna, Austria: R Foundation for Statistical Computing Available online at: http://www.R-project.org/

[B36] Rechenchoski D. Z. Marim A. Dambrozio L. Carolina A. Vivan P. Schuroff P. A. (2017). Antimicrobial activity evaluation and comparison of methods of susceptibility for *Klebsiella Pneumoniae* carbapenemase (KPC)-producing *Enterobacter* spp. isolates. *Braz. J. Microbiol.* 48 509–514. 10.1016/j.bjm.2017.01.008 28552659 PMC5498445

[B37] Redgrave L. S. Sutton S. B. Webber M. A. Piddock L. J. V. (2014). Fluoroquinolone resistance: mechanisms, impact on bacteria, and role in evolutionary success. *Trends Microbiol.* 22 438–445. 10.1016/j.tim.2014.04.007 24842194

[B38] Royal D. (2016). Belgian royal decree of 21 July 2016 concerning the conditions for the use of medicines by veterianarians and by the responsible for the animals. *Belg. Staatsblad.* 203 46569–46586.

[B39] Shrout P. E. Fleiss L. J. (1979). Intraclass correlations: uses in assessing rater reliability. *Psychol. Bullet.* 86 420–428. 10.1037/0033-2909.86.2.420 18839484

[B40] Temmerman R. Antonissen G. Vanantwerpen G. Vanrobaeys M. Haesebrouck F. Garmyn A. (2020). *Evaluation of Fluoroquinolone Resistance in Clinical Avian Pathogenic Escherichia Coli Isolates from Flanders.* Lausanne, Switzerland: Frontiers in Veterinary Science. 10.3390/antibiotics9110800 PMC7696922 33198103

[B41] Thornsberry C. Anhalt J. P. Washington J. A. Mccarthy L. R. Schoenknecht F. D. Sherris J. C. (1980). Clinical laboratory evaluation of the abbott ms-2 automated antimicrobial susceptibility testing system: report of a collaborative study. *J. Clin. Microbiol.* 12 375–390. 10.1128/jcm.12.3.375-390.1980 6783678 PMC273593

[B42] Trouchon T. Lefebvre S. (2016). A Review of enrofloxacin for veterinary use. *Open J. Vet. Med.* 06 40–58. 10.4236/ojvm.2016.62006

[B43] WHO Advisory Group on Integrated Surveillance of Antimicrobial Resistance (AGISAR). (2019). Critically Important Antimicrobials for Human Medicine 6th Re.Vision 2018. Ranking of Medically Important Antimicrobials for Risk Management of Antimicrobial Resistance Due to Non-Human Use. Available online at: https://apps.who.int/iris/bitstream/handle/10665/312266/9789241515528-eng.pdf?ua=1 (accessed December 19, 2019).

